# Evaluation of Physiochemical and Biological Properties of Biofunctionalized Mg-Based Implants Obtained via Large-Scale PEO Process for Dentistry Applications

**DOI:** 10.3390/jfb14070338

**Published:** 2023-06-27

**Authors:** Julia Radwan-Pragłowska, Łukasz Janus, Tomasz Galek, Ernest Szajna, Aleksandra Sierakowska, Karol Łysiak, Mirosław Tupaj, Dariusz Bogdał

**Affiliations:** 1 Department of Biotechnology and Physical Chemistry, Faculty of Chemical Engineering and Technology, Cracow University of Technology, Warszawska 24 Street, 31-155 Cracow, Polanda.sierakowska3530@doktorant.pk.edu.pl (A.S.); dariusz.bogdal@pk.edu.pl (D.B.); 2 Faculty of Mechanics and Technology, Rzeszow University of Technology, Kwiatkowskiego 4 Street, 37-450 Stalowa Wola, Poland; t.galek@prz.edu.pl (T.G.); k.lysiak@prz.edu.pl (K.Ł.); mirek@prz.edu.pl (M.T.); 3 WEA Techlab Sp. z o. o., Perla 10, 41-301 Dabrowa Gornicza, Poland; e.szajna@uce.com.pl

**Keywords:** dental biomaterials, bone tissue replacement, structural properties

## Abstract

An increasing number of tooth replacement procedures ending with implant failure generates a great need for the delivery of novel biomedical solutions with appropriate mechanical characteristics that would mimic natural tissue and undergo biodegradation. This phenomenon constitutes a significant difficulty for scientists, since currently applied biomaterials dedicated for this purpose are based on stainless steel, Ti, and Ti and CoCr alloys. One of the most promising raw materials is magnesium, which has been proven to promote bone regeneration and accelerate the tissue healing process. Nevertheless, its high reactivity with body fluid components is associated with fast and difficult-to-control biocorrosion, which strongly limits the application of Mg implants as medical devices. The achievement of appropriate functionality, both physiochemical and biological, to enable the commercial use of Mg biomaterials is possible only after their superficial modification. Therefore, the obtainment of uniform, reproducible coatings increasing resistance to the aqueous environment of the human body combined with a nanostructured surface that enhances implant–cell behaviors is an extremely important issue. Herein, we present a successful strategy for the modification of Mg implants via the PEO process, resulting in the obtainment of biomaterials with lower corrosion rates and superior biological properties, such as the promotion of extracellular matrix formation and a positive impact on the proliferation of MG-63 cells. The implants were investigated regarding their chemical composition using the FT-IR and XRD methods, which revealed that MgO layer formation, as well as the incorporation of electrolyte components such as fluorine and silica, were responsible for the increased microhardness of the samples. An extensive study of the biomaterials’ morphology confirmed that successful surface modification led to a microporous structure suitable for the attachment and proliferation of cells. The three-layer nature of the newly-formed coatings, typical for PEO modification, was confirmed via cross-section analysis. A biocorrosion and biodegradation study proved that applied modification increased their resistance to body fluids. The cell culture study performed herein confirmed that the correct adjustment of modification parameters results in a lack of cytotoxicity of the magnesium implants, cell proliferation enhancement, and improvement in extracellular matrix formation.

## 1. Introduction

Bone is one of the most often replaced body tissues, and constantly undergoes remodeling [1,2]. Although there has been great progress in the fields of tissue engineering and regenerative medicine, bones, due to their specific characteristics, especially in terms of mechanical properties such as Young’s modulus and density, which are crucial as they have supporting functions in the human body, are very difficult to properly replace, especially using common nanofibrous or hydrogel scaffolds. Therefore, for many years, metallic biomaterials have been superior to polymeric and ceramic ones for this application [1,2,3]. Their field of application in biomedicine is very broad, since they may be used not only as screws, plates, nails, and clamps, but also as hip, knee, and shoulder joints and in arthroplasty, maxillofacial surgery, and dentistry (dental and orthodontic implants, internal fixation). Metallic biomaterials can be applied as temporary implants (rods, nails, wires, screws, joining plates, etc.) prepared mostly from chrome–nickel steels which must be removed after a fixed period of time. These are used for, e.g., healing a broken bone or as permanent implants such as joint prostheses, artificial heart valves, wires, components of pacemakers and nerves, or surgical sutures [4,5]. For this purpose, titanium and its alloys, as well as cobalt/chromium alloys, are considered to be the most popular, mostly due to their chemical inertness and high tensile strength [6]. Generally, metallic biomaterials are characterized by superior mechanical durability, especially compared to polymeric ones, yet under in vivo conditions they may react with components of human body fluids, leading to numerous undesired effects due to the toxic nature of the metal ions being released [4,7]. The most common implant–bone interactions include redox reactions at the interface (electron exchange), hydrolysis of corrosion products (proton exchange), and, finally, the formation of metal–organic complexes. Consequently, their mediocre chemical stability after implementation, causing undesired host tissue responses such as inflammatory reactions or pain, leads to reoperation in many cases. Therefore, currently, corrosion resistance (potentiodynamic characteristics), chemical composition, the level of non-metallic inclusions, phase composition, and mechanical properties are the main criteria for the selection of metals for dentistry implants [5].

Nowadays, the very rapid progress of regenerative medicine, together with the development of techniques enabling tissue engineering, is drawing attention to fully biodegradable materials characterized by mechanical durability which enable new bone tissue formation and maturation. Unfortunately, such characteristics are not yet reachable for standard metals or their alloys which are applied in dentistry. Taking under consideration our aging society, increasing life expectancy, dynamic medicine progress, and desire to increase comfort in life, the development of novel metallic biomaterials which would meet the criteria of not only biocompatibility, but also bioactivity and biodegradability, is of the highest importance [2,8,9].

Currently, magnesium constitutes the most relevant and promising material that can used for the preparation of metallic implants due to its superiority, which results from its susceptibility to biodegradation under in vivo conditions [10,11]. This element is not involved in metabolic reactions. Contrary to other metals that are applied in dentistry/oral surgery, the choice of Mg-based biomaterials avoids post-implant revision surgery. It can be applied for the fabrication of orthopedic implants such as plates, rods, screws, and cardiovascular stents. Importantly, compared to steel or titanium, Mg is characterized by mechanical properties which mimic the Young’s modulus and density of human bone in a far superior manner [12,13]. Moreover, based on cellular and molecular studies of magnesium alloy-based implants during the early stages of healing, it has been proven that the release of magnesium ions promotes rapid bone formation. Furthermore, it activates osteogenic signals in the vicinity of the implants in patients with symptoms of osteoporosis. Magnesium is another relevant element in human body, and it belongs to the group of elements of fundamental biological importance. Therefore, it may act not only as a mechanical support, but also as a scaffold for new bone tissue formation, and should be considered as a raw material for the most promising metallic implants [14,15]. However, the high reactivity of pure magnesium strongly limits the commercial use of Mg-based implants due to their insufficient resistance to biodegradation under in vivo conditions [16,17,18].

Diverse procedures to obtain Mg alloys and modify the Mg surface have been reported, aiming to overcome the problem of the too-low biocorrosion resistance of pure magnesium [13,19]. These include chemical conversion coating, electrophoretic deposition, and laser treatment [20,21]. Acceptable levels of implant protection can be achieved by combining magnesium and other elements, resulting in the formation of various alloys [22,23]. However, such materials are characterized by rather high structural heterogeneity, making them susceptible to various types of corrosion, especially crevice and pitting types. Therefore, plasma electrolytic oxidation (PEO) is the most successful approach, as it is able to achieve fast, facile, and efficient modification of implant surfaces due to the formation of thick and dense MgO layers, and it can provide complete sample coverage [24,25]. Nevertheless, data are lacking regarding such layers’ full characteristics. Such data would include cross-sections providing knowledge of their thickness and uniformness values, or microstructure studies [26], which are crucial in order to gain complete information on the potential stability of materials in the human body environment and the expected cellular behaviors at the implant–cell interface [27,28].

Herein, we report the successful obtainment of biodegradable Mg-based biomaterials characterized by significantly increased functionality. The goal was achieved by performing PEO modification. The novelty of the paper is that it covers obtainment of the magnesium implants via plasma electrolytic oxidation using industrial-scale equipment and extensive evaluation of the impact of modification parameters on the physicochemical, morphological, and biological properties of the final products. Biomaterials were investigated for their chemical structures, and their surfaces were extensively evaluated. This was followed by a biocorrosion and biodegradation study, which demonstrated their superiority to commercially used Ti dental implants. The positive results of the cell culture study carried out on the MG-63 cell line, together with the confirmation of bioactivity of the prepared implants, revealed their enhanced, high potential in dentistry application, especially in terms of temporary replacement of bone tissue.

## 2. Materials and Methods

### 2.1. Obtainment of Implants

The modification of the magnesium implants was carried out via plasma electrolytic oxidation (PEO), applying a current density of 150 mA/cm^2^. As a starting material, a magnesium cylinder was used. Before electrochemical oxidation, it was divided into samples of 1.9 mm diameter and 5 mm height. In the next step, each of them was drilled and threaded. To provide uniform coverage, the surfaces were pre-treated using sandpaper. Before the modification, to provide appropriate purity, samples were subjected to ultrasound treatment with (15 min) and without (5 min) a detergent. The smoothened implants were immersed in the electrolyte containing NaOH (5 g/L), NH_4_F—5 g/L, and Na_2_SiO_3_ × 5H_2_O (25 g/L). Both the electrolyte and anodization parameters were chosen based on the preliminary study and the observation of silvering, confirming plasma excharges. Pre-treated samples were subjected to an anodization process according to the parameters (voltage, current) given in Table 1.

### 2.2. Characterization of Materials

Spectroscopic analysis (FT-IR) was performed using a Thermo Nicolet Nexus 470 FT-IR spectrometer equipped with an ATR adapter. To demonstrate the increased resistance of the magnesium implants obtained by electrolytic plasma oxidation, biocorrosion tests in simulated body fluids and electrochemical corrosion measurements were performed. The samples of magnesium implants were placed in 50 mL of a solution of simulated body fluids (SBF) at a temperature of 37 °C for a period of seven days. During incubation, the surface and metallic core of the magnesium core biocorroded, resulting in the formation of a white magnesium hydroxide precipitate and the release of hydrogen gas bubbles. The obtained precipitate was separated from the magnesium implant and the concentration of released magnesium ions was determined. The precipitate was dissolved in concentrated nitric acid, and the concentration of magnesium ions was determined using atomic absorption spectroscopy (AAS). The electrochemical analysis was carried out using a three-electrode system consisting of a saturated calomel reference electrode, a working electrode in the form of a platinum wire, and the tested metallic magnesium implant sample. The electrochemical corrosion test was performed using the potential of the working electrode in a range from minus −2 V to plus +1 V, with a scanning rate of 20 mV/min. The obtained curves were analyzed using the Tafel method to determine the values of the biocorrosion current and the potential.

The morphology studies were performed using a high-resolution scanning electron microscope (SEM, Tescan). To obtain microphotographs, the samples were sputtered with copper. Porosity was determined using Fiji Image J software, 2023 version.

Hardness was measured using ATM Qness 60 M EVO hardness tester (Vickers method), in accordance with the PN-EN ISO 6507-1 norm [29]. The test was carried out with a load of 5 kgf. Five measurements were taken for each sample.

Cytotoxicity tests were carried out using the MG-63 cell line according to ISO 10993 standard [30]. The cells were cultured under standard conditions (5% CO_2_, 37 °C, 95% humidity) in DMEM medium supplemented with antibiotic/antimycotic solution and FBS. The medium was changed every 48 h. Cell morphology was studied using an inverted optical microscope. All reagents were purchased from SigmaAldrich, Poznań, Poland.

## 3. Results and Discussion

**Figure 1:**
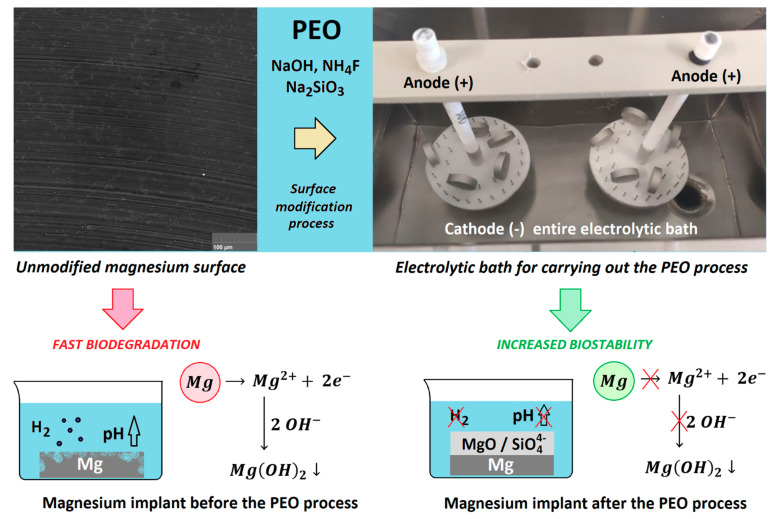
Mg implants modification pathway.

Figure 1 provides a general overview of our work. It shows an HR-SEM microphotograph of an unmodified magnesium surface and its modification pathway, resulting in implant coating and potential resistance to improvements in biocorrosion. PEO modification enables surface passivation, leading to a significant increase in biocompatibility and stability in the environment of the body. Moreover, contrary to many other techniques, it has the capability to include full sample coverage, preventing crevice corrosion. As shown in Figure 1, the process performed herein resulted in macroscopic changes to the samples’ surfaces. All of the obtained implants were further used for the experiments described in the following sections.

**Figure 2:**
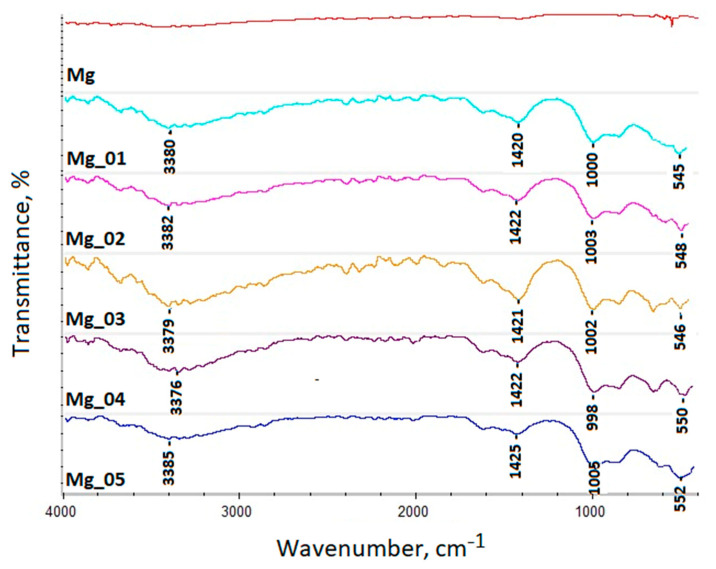
FT-IR spectra of the pure magnesium and modified samples (native magnesium—red, Mg 200 V—blue, Mg 225 V—pink, Mg 235 V—yellow, Mg 250 V—purple, and Mg 260 V—indigo).

Firstly, the chemical structures of the modification products were analyzed to verify changes in surface composition and to determine whether the PEO process performed successfully. Figure 2 presents the results of the FT-IR analysis of unmodified magnesium and the implants after PEO modification. The spectrum of native Mg exhibited no bands. As can be observed, the application of high voltage resulted in the formation of new bands, confirming successful passivation due to plasma exchanges under high voltage. All of them revealed bands characteristic of magnesium oxide at 545 cm^−1^ (sample 1), 548 cm^−1^ (sample 2), 546 cm^−1^, (sample 3), 550 cm^−1^ (sample 4), and 552 cm^−1^ (sample 5), respectively, which correspond to the reports of other researchers. Additionally, bands confirming Mg_2_SiO_4_ incorporation were noticed at 1000 cm^−1^ (sample 1), 1003 cm^−1^ (sample 2), 1002 cm^−1^ (sample 3), 998 cm^−1^ (sample 4), and 1005 cm^−1^ (sample 5), respectively.

Moreover, all of the treated substrates exhibited bands of low intensity at 3380 cm^−1^ (sample 1), 3382 cm^−1^ (sample 2), 3379 cm^−1^ (sample 3), 3376 cm^−1^ (sample 4), and 3385 cm^−1^ (sample 5). These came from of H–O–H, and can be attributed to water molecules. This phenomenon is typical for magnesium oxide. Therefore, the band intensity can be correlated with the MgO layer’s thickness. Similarly, the bands reaching the maximum at 1420 cm^−1^ (sample 1), 1422 cm^−1^ (sample 2), 1421 cm^−1^ (sample 3), 1422 cm^−1^ (sample 4), and 1425 cm^−1^ (sample 5) can be categorized by the fact that H_2_O adsorbed to the metal oxide surface, as well as the fact that C=O came from CO_2_. It can be noticed that all of the bands’ intensities vary between the samples, which can be correlated with the MgO coating thickness.

**Figure 3:**
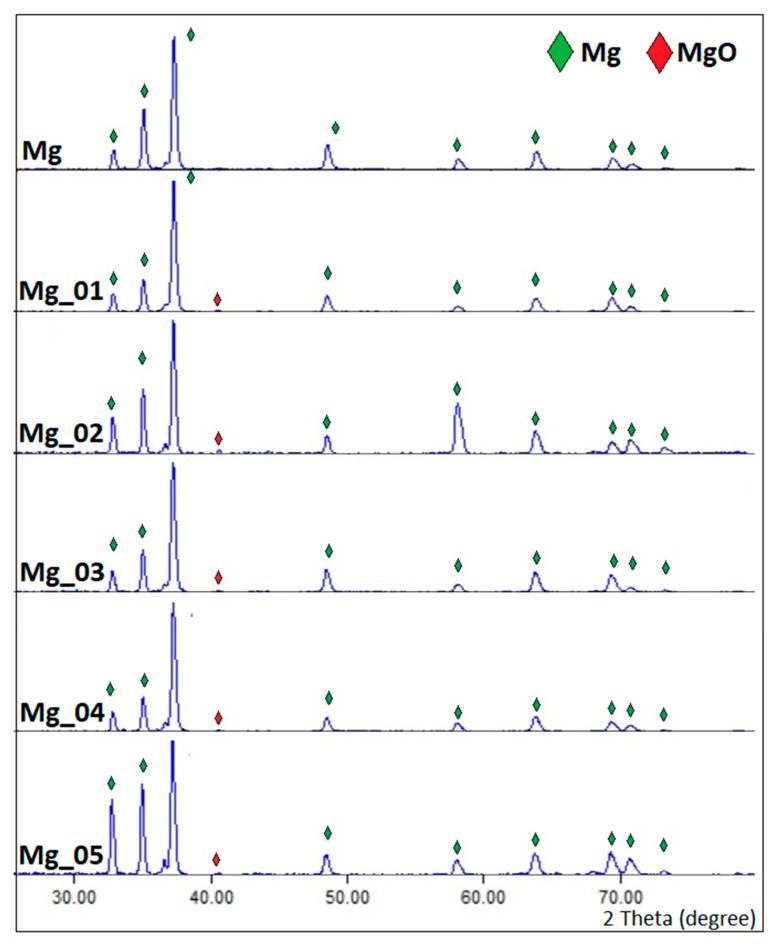
XRD spectra of the pure magnesium and modified samples.

It is known that PEO modification leads to superficial oxidation, resulting in the formation of three-layered structures. Such phenomena occur due the plasma chemicals and the chemicals interacting with oxygen thermal diffusion. To further investigate superficial changes to the magnesium implants, XRD analysis was carried out. The results are given in Figure 3. It revealed that all samples contained pure magnesium, which was typical for Mg patterns [31,32]. However, as shown in Figure 2, samples Mg_01–Mg_05 were composed of two extra phases, namely, magnesium oxide, which can be proven by the presence of patterns typical for MgO above 40.00, and Mg_2_SiO_4_, coming from electrolytes in a changed form (Na_2_SiO_3_ × 5H_2_O). In addition, the results shown in Figure 3 indicate that the newly formed oxide layers are relatively thin.

**Figure 4:**
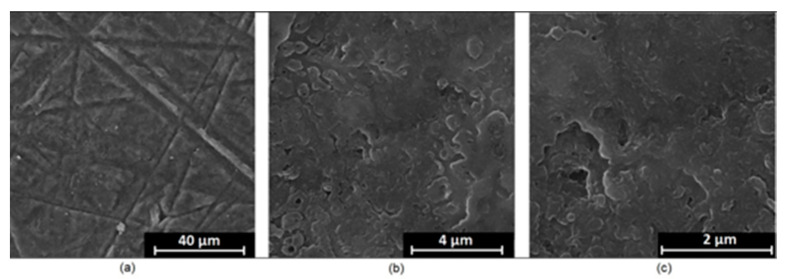
SEM microphotographs of the Mg_01 sample: (**a**)—10,000× magnification; (**b**)—100,000× magnification; (**c**)—250,000× magnification.

**Figure 5:**
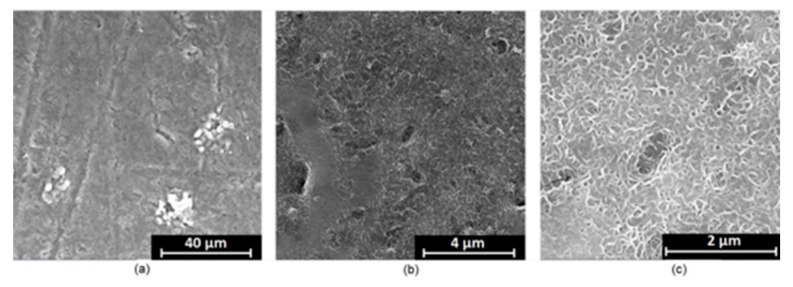
SEM microphotographs of the Mg_02 sample: (**a**)—10,000× magnification; (**b**)—100,000× magnification; (**c**)—250,000× magnification.

**Figure 6:**
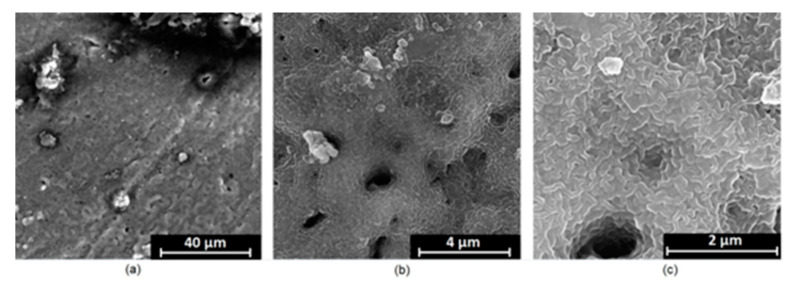
SEM microphotographs of the Mg_03 sample: (**a**)—10,000× magnification; (**b**)—100,000× magnification; (**c**)—250,000× magnification.

**Figure 7:**
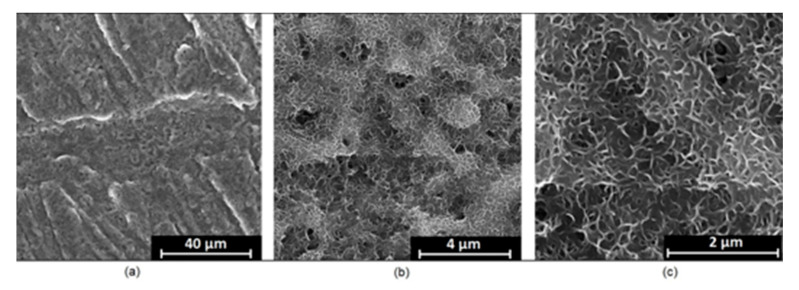
SEM microphotographs of the Mg_04 sample: (**a**)—10,000× magnification; (**b**)—100,000× magnification; (**c**)—250,000× magnification.

**Figure 8:**
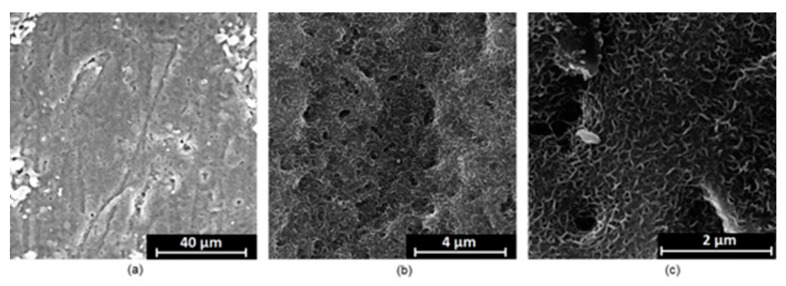
SEM microphotographs of the Mg_05 sample: (**a**)—10,000× magnification; (**b**)—100,000× magnification; (**c**)—250,000× magnification.

It is well known that the biomaterial surface is crucial for proper interface–cell interaction. Adequate porosity of the material enhances adhesion, spreading, and extracellular matrix formation, thus supporting new tissue formation. On the other hand, uneven and rough biomaterials may cause irritation or even damage to the surrounding tissues during implantation. As shown in Figure 1, unmodified magnesium implants are characterized by their smooth, uniform structure, which do not favor cell attachment. Figure 4, Figure 5, Figure 6, Figure 7 and Figure 8 show surface morphology after PEO modification under different parameters (applied voltage). It can be observed that all samples exhibit “pancake”-like structures, typical for plasma electrolytic oxidation, containing discharge channels as a consequence of the plasma discharge [33]. Importantly, during the process, liquid substances flow through them and cool down to solid form. Therefore, visible boundaries between the structures can be spotted. As one may notice, all samples exhibited high porosity and well-developed surfaces, which are an extremely desired features for biomaterials dedicated to the support of new tissue formation. Such a morphology enables cell attachment, adhesion, spreading, and proliferation. While unmodified magnesium exhibits smooth, solid surface with no pores, SEM images revealed that depending on the parameters, different sample porosity levels were achieved as a result of PEO treatment, namely, 41.43% (SD = 24.98) for the Mg_01 sample, 40.28% (SD = 49.17) for the Mg_02 sample, 41.36% (SD = 46.82) for the Mg_03 sample, 41.70 (SD = 45.87) for the Mg_04 sample, and 41.77 (SD = 40.09) for the Mg_05 sample, respectively. This morphology enhances extracellular matrix formation. It can be noticed that the sizes of the superficial voids differ between the samples, and can be correlated with the voltage applied during the PEO process, since its increase resulted in a deeper penetration of molten phases as well as more powerful discharge. Interestingly, it can be noted that samples vary in terms of their surface homogeneity. However, all of them exhibit pores at both the micro- and nanoscales. This is an important feature during tissue formation in three dimensions, since microarchitecture plays a crucial role during metallic scaffold replacement with new bone.

Apart from the appropriate microarchitecture, for proper fixation and osseointegration, another critical parameter is the chemical composition of the material. Magnesium is an element which occurs in the human body naturally and positively affects processes associated with bone regeneration. However, due to its high chemical reactivity, to be more suitable for biomedical application, it must undergo modification. PEO requires the use of an electrolyte. The correct choice of its composition is crucial, since, due to the nature of electrochemical processes, they are incorporated into the material’s structure permanently, as shown in Figure 2 and Figure 3 [33,34].

**Figure 9:**
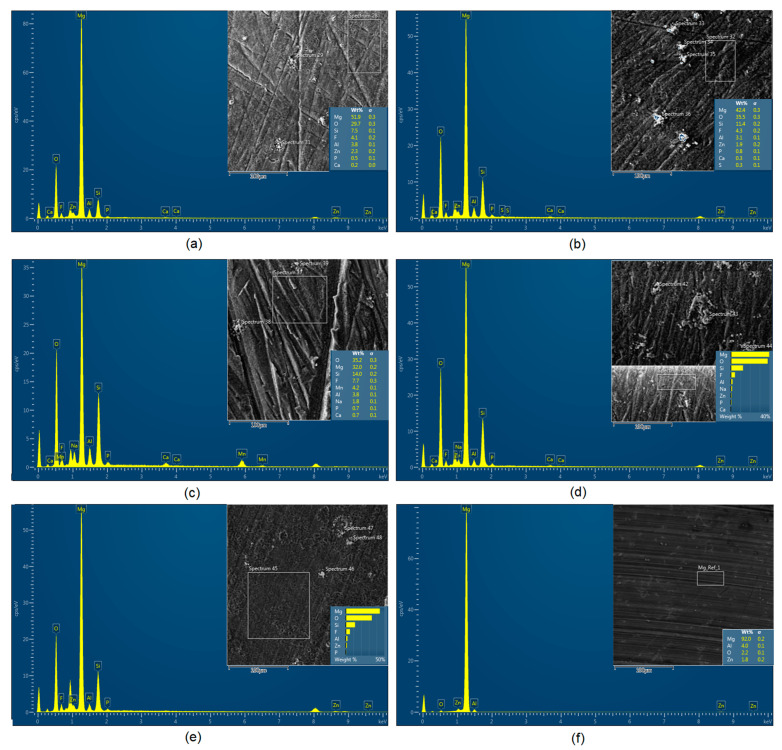
Surface elemental composition (XRF): (**a**)—Mg_01; (**b**)—Mg_02; (**c**)—Mg_03; (**d**)—Mg_04; (**e**)—Mg_05; (**f**)—Mg [35].

**Figure 10:**
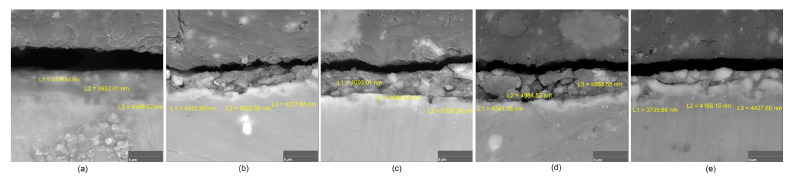
Sample cross-sections: (**a**)—Mg_01; (**b**)—Mg_02; (**c**)—Mg_03 (**d**)—Mg_04; (**e**)—Mg_05.

Although magnesium-based implants and other biomaterials are biosafe and even stimulate the regeneration process, it must be acknowledged that they undergo rapid biodegradation, which is very random in terms of speed rate, because of chemical reactions with human body fluids. Consequently, gaseous hydrogen and Mg(OH)_2_ are generated, leading to a significant increase in local pH. Therefore, some undesired side effects can occur, and the newly formed, histologically immature tissue may be damaged. Therefore, successful modification of Mg biomaterials leading to an increase in biocorrosion resistance and stability in body fluids is an issue of the greatest importance. The PEO process is known to be the most promising method for this purpose, since is considerably easy, accessible, and low-cost, thus enabling the fabrication of modified materials on a large scale. Moreover, contrary to many other methods, it provides durable surface modification due to the chemical phenomena occurring during PEO performance. Generally, during the process, three different layers are being formed: an external one, which is usually highly porous and somewhat compact, as shown in Figure 4, Figure 5, Figure 6, Figure 7 and Figure 8; the middle one, which is very dense and homogeneous, and is mostly responsible for protection of the substrate from the environment and mechanical wear; and the internal layer, which can also be described as dense, although it is thin and sticks to the modified material surface, mostly determining corrosion resistance [32]. Figure 9 shows a surface elemental composition analysis of the obtained samples, which was performed by the XRF method. All implants exhibited the presence of pure magnesium and trace amounts of impurities of raw materials such as Al, Zn, Mn. Noteworthily, in the case of modified samples, there was a significant increase in the amount of oxygen, confirming the formation of a passivation layer (MgO) due to the performance of the PEO process. Notably, it can be noted that the Mg/O ratio significantly varied between the samples, which suggests different thicknesses of the magnesium oxide coating depending on the PEO parameters. Additionally, new elements such as Si and F, former electrolyte components, were present; they were incorporated into the material structure during plasma exchanges when high voltage was applied. No toxic impurities were spotted. The aforementioned results correspond to the data presented in Figure 2 and Figure 3. Knowledge of coating thickness is a crucial parameter in terms of potential biomedical applications, since it determines its stability and durability. Figure 10 reveals cross-sections of the modified samples. As can be observed, all of the specimens exhibited three-layered structures. The average coating thickness varied between 5–6 μm. The thickest layers were obtained for the Mg_03 and Mg_04 samples. In all cases, the middle layer had the highest diameter, except for sample Mg_01, where the outer layer could be distinguished as the thickest one. The barrier layers also differed between the samples; the thickest were those of the Mg_3, Mg_04, and Mg_05 sample. Taking all of these data together, based on the thickness and density of each layer, the Mg_03 and Mg_04 samples showed the most promising cross-section characteristics.

**Figure 11:**
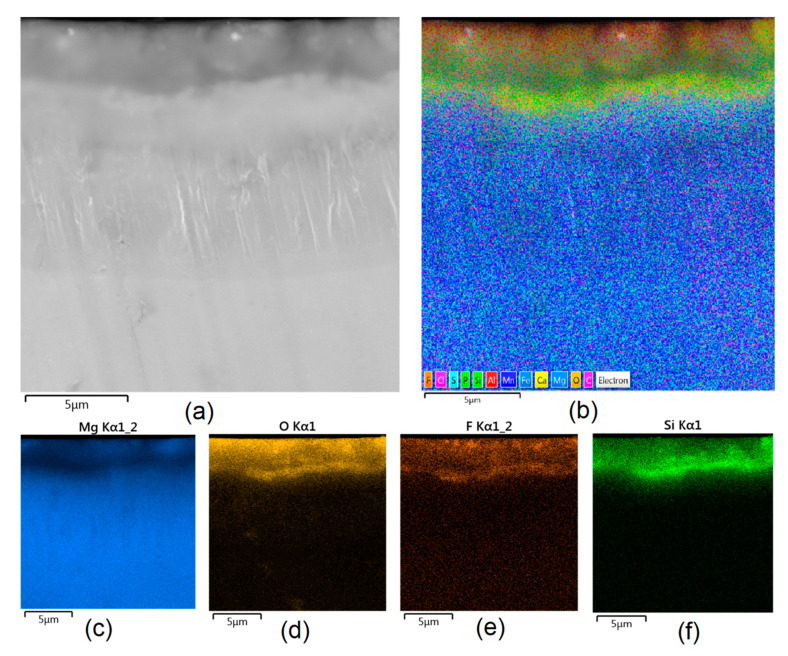
Elemental analysis of Mg_01 sample cross-section: (**a**)—general appearance, 50,000× magnification; (**b**)—surface mapping (all elements); (**c**)—magnesium; (**d**)—oxygen; (**e**)—fluorine; (**f**)—silica.

**Figure 12:**
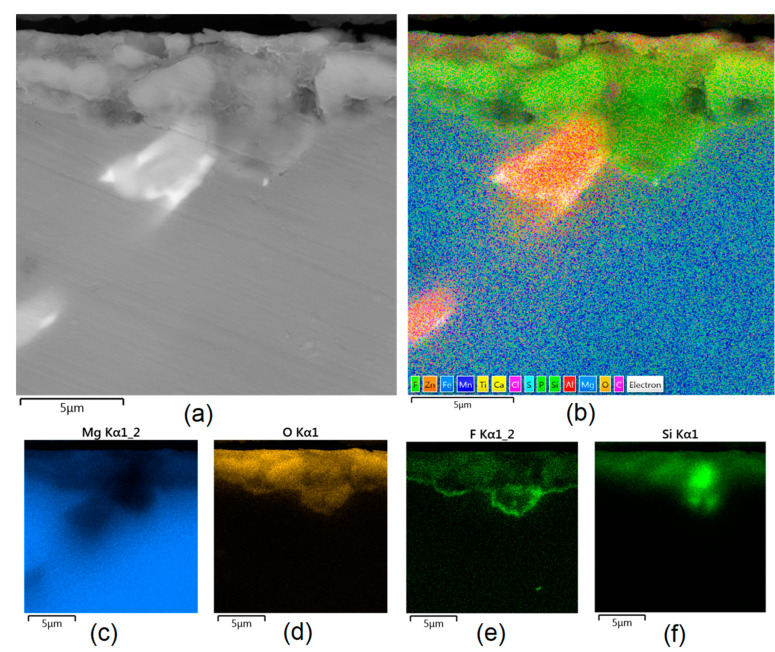
Elemental analysis of Mg_02 sample cross-section: (**a**)—general appearance, 50,000× magnification; (**b**)—surface mapping (all elements); (**c**)—magnesium; (**d**)—oxygen; (**e**)—fluorine; (**f**)—silica.

**Figure 13:**
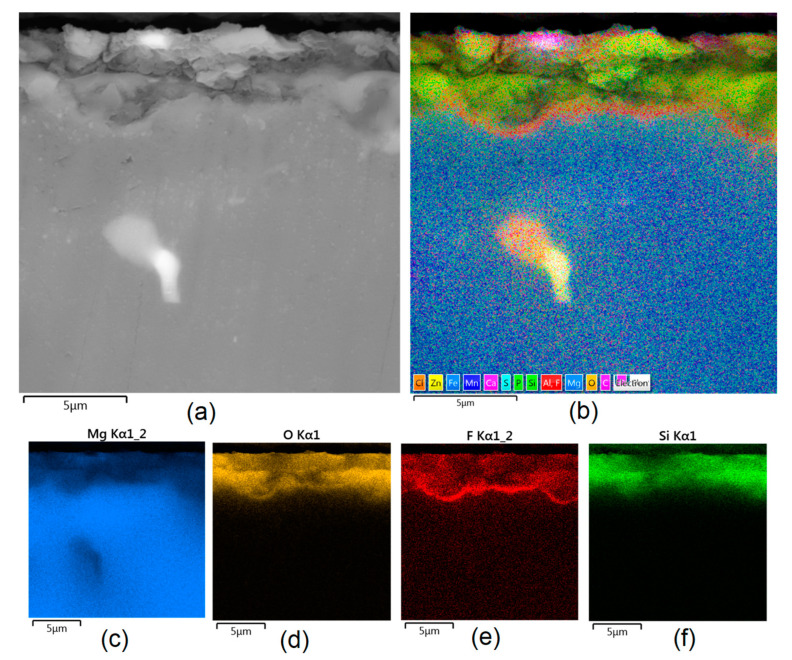
Elemental analysis of Mg_03 sample cross-section: (**a**)—general appearance, 50,000× magnification; (**b**)—surface mapping (all elements); (**c**)—magnesium; (**d**)—oxygen; (**e**)—fluorine; (**f**)—silica.

**Figure 14:**
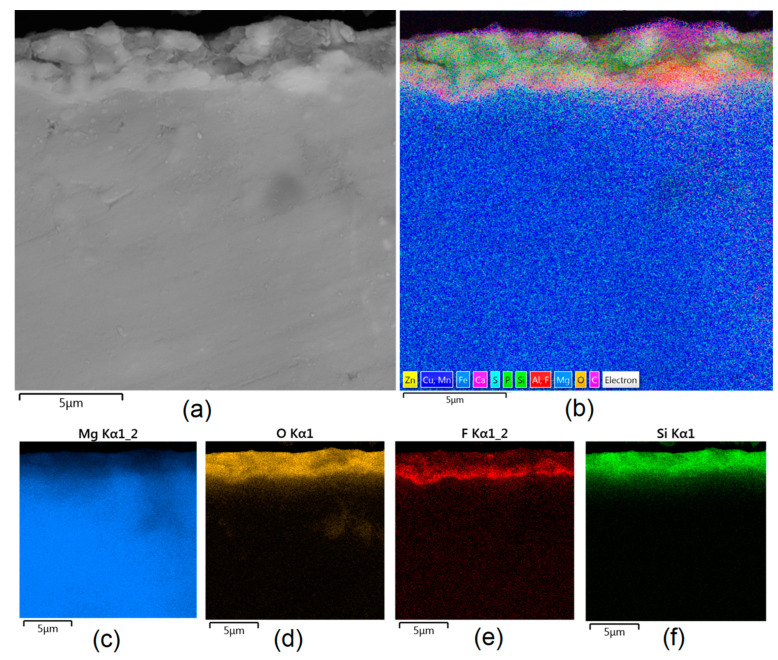
Elemental analysis of Mg_04 sample cross-section: (**a**)—general appearance, 50,000× magnification; (**b**)—surface mapping (all elements); (**c**)—magnesium; (**d**)—oxygen; (**e**)—fluorine; (**f**)—silica.

**Figure 15:**
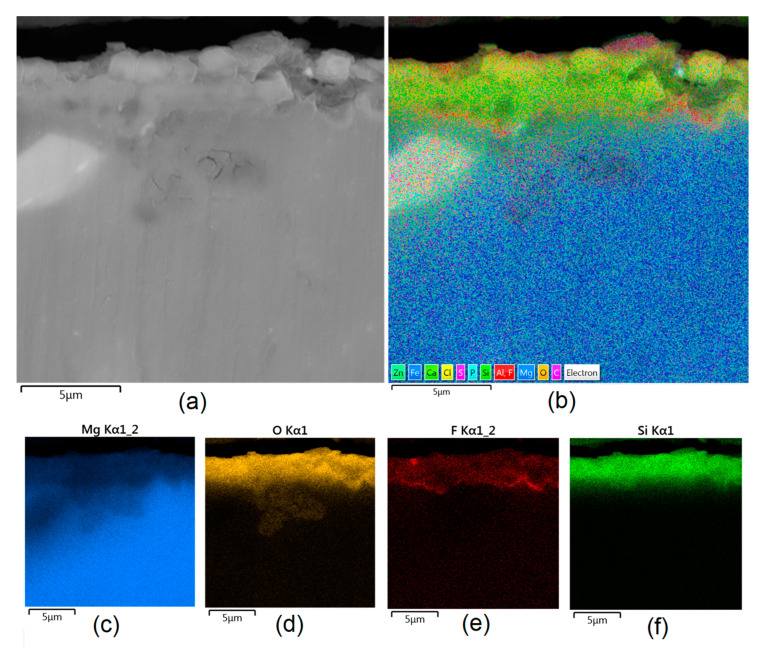
Elemental analysis of Mg_05 sample cross-section: (**a**)—general appearance, 50,000× magnification; (**b**)—surface mapping (all elements); (**c**)—magnesium; (**d**)—oxygen; (**e**)—fluorine; (**f**)—silica.

Apart from the layer’s thickness, another interesting parameter is chemical composition. As previously discussed, the PEO process is characterized by the phenomenon of migration of electrolyte components through the material surface, possibly ending with them being permanent built in. As shown in Figure 11, Figure 12, Figure 13, Figure 14 and Figure 15, in all cases, there were significant differences in chemical composition. This provides insight into the process mechanism and, without a doubt, confirms surface modification. There was a very visible, distinctive layer of MgO on pure Mg. Moreover, the figures clearly show the three layers of the coating, which differ in their elemental compositions. In all cases, the coatings contained oxygen and magnesium, confirming the occurrence of an oxidation process. Moreover, large amounts of silica can be also observed. These originated from Mg_2_SiO_4_, which migrated through the channels formed during plasma discharge. The most uniform distribution was obtained for samples Mg_03, Mg_04, and Mg_5. One may observe that fluorine accumulation is correlated with the voltage being applied. The higher the voltage, the lower the occupation of the material, which can be explained by the fact that F-ions are highly mobile in electric fields due to their electronegative nature. In the case of sample Mg_01 (200 V), fluorine was present throughout all three layers, whereas for the other four samples (225 V, 235 V, 250 V, 260 V) it mainly accumulated in the barrier layer. Additionally, some elemental inclusions (Zn, Al) could be observed in the Mg_02, Mg_03 and Mg_05 samples. Elemental analysis of cross-sections of the samples also shows that the most homogeneous coating was achieved in the case of the Mg_04 sample (250 V).

**Figure 16:**
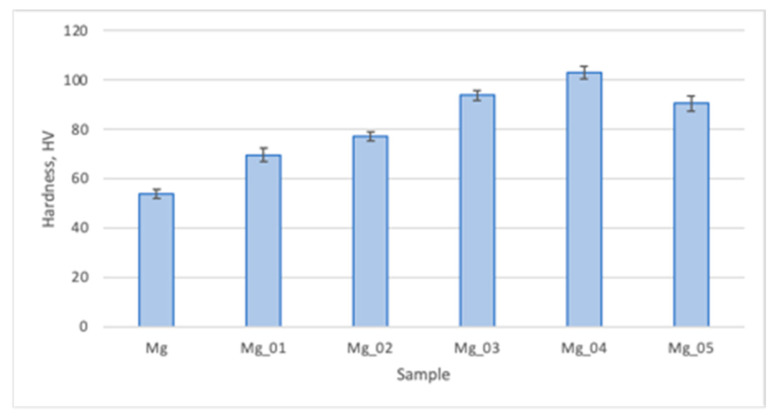
Microhardness (Vickers method) of the evaluated samples.

Pure magnesium is known for its resemblance, in terms of mechanical properties, to natural bone. As shown in Figure 16, the hardness of native Mg is around 54 HV, which corresponds to healthy bone. However, it is known that this parameter varies between cancellous or compact bones and their types. Moreover, hardness provides insight into the tissue quality. As shown in Figure 16, the modifications which we performed resulted in a significant increase in microhardness which was correlated with the applied voltage, excluding the Mg_05 sample. This phenomenon can be explained by the formation of a dense layer of magnesium oxide. Moreover, the addition of silica positively affected this parameter. Figure 16 shows that the Mg_05 sample’s HV was lower than not only Mg_04, but also Mg_03, which suggests that increasing the voltage during the PEO process helps to improve the biomaterial performance only to a point. Noteworthily, excessively high hardness may be associated with a risk of the materials cracking during loading. When comparing all of the samples, it can be observed that applying the appropriate voltage and choosing the correct electrolyte provides the possibility of obtaining a biomaterial with the desired durability. The highest microhardness was revealed for sample 4, and cross-sectional analysis revealed that its newly formed oxygen layer was the thickest, most compact, and most uniform. Therefore, it may be assumed that the proposed biomaterials can replace damaged or lost bone due to its mechanical functionality, providing appropriate support for other tissues. In addition, our results show that the prepared samples can be used to replace types of bones which differ in terms of microhardness and microstructure.

One of the crucial parameters for metallic biomaterials dedicated for medical application is their resistance to biocorrosion [36]. Generally, there are three major factors which determine this property. These include chemical composition, microstructure, and coating presence. Mechanical durability is also important, since structural disturbances such as cracks or hollows significantly increase susceptibility to corrosion.

**Figure 17:**
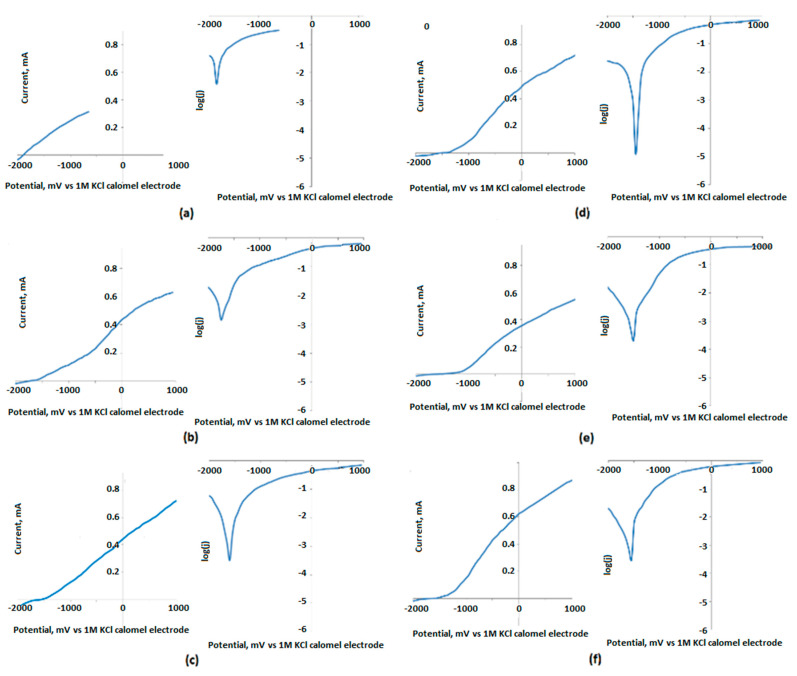
Biocorrosion study of the samples: (**a**)—Mg; (**b**)—Mg_01; (**c**)—Mg_02; (**d**)—Mg_03; (**e**)—Mg_04; and (**f**)—Mg_05.

**Figure 18:**
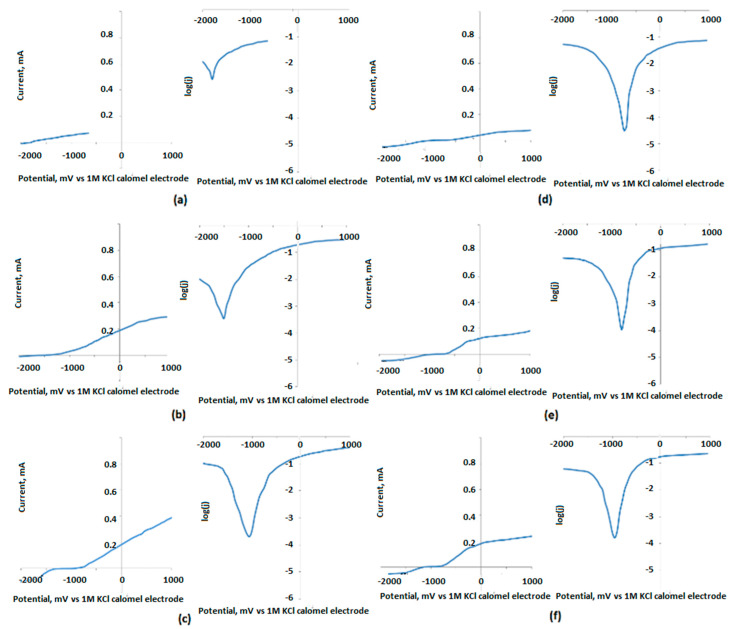
Biocorrosion study of samples (**a**)—Mg; (**b**)—Mg_01; (**c**)—Mg_02; (**d**)—Mg_03 (**e**)—Mg_04; and (**f**)—Mg_05 after 7 days of incubation in SBF.

**Figure 19:**
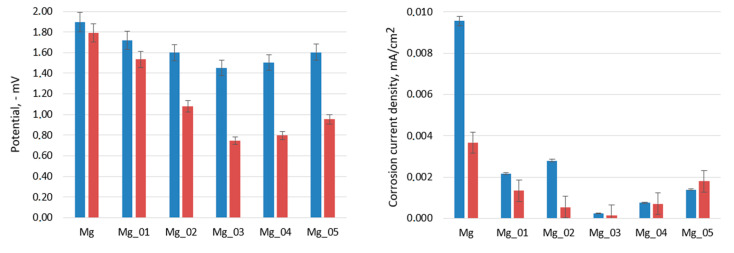
Left—potential of the samples before (blue) and after incubation with SBF solution for 7 days at 37 °C (red); right—current density of the samples before (blue) and after incubation with SBF solution for 7 days at 37 °C (red).

To characterize the surface resistance of the obtained biomaterials to corrosive agents, electrochemical tests were carried out. For this purpose, a measuring system consisting of three electrodes was prepared. These included a working electrode, which was a sample of a magnesium implant; a reference electrode, in the form of a saturated calomel electrode; and a platinum wire as a counter electrode. Figure 17, Figure 18 and Figure 19 show the results of the biocorrosion study. The experiment was carried out on pure magnesium, and samples Mg_01–Mg_05 were obtained. The results were repeated after 7 days of incubation in SBF at 37 °C. Pure magnesium metal has a very low potential due to its very high chemical reactivity. Along with the chemical modification of the surface leading to the formation of a magnesium oxide passivate layer, silicate, and magnesium fluoride, the potential of the electrode shifted towards a positive value.

As expected, it can be clearly demonstrated that the unmodified Mg sample is characterized by a significantly higher corrosion rate. The results revealed that the formation of three-layered coatings composed of MgO and some additives successfully hampers the migration of simulated body fluid components, constituting an effective barrier between pure Mg and H_2_O and Cl^−^ ions. Biocorrosion resistance can be correlated with voltage applied during PEO modification. The obtained results correspond to other researchers’ data [37,38]. It must be mentioned that providing appropriate conditions for apatite/hydroxyapatite deposition on the substrates has a synergistic effect, since, after incubation with SBF, the potential and corrosion current density are lower (excluding the Mg_05 sample). During the electrochemical analysis of the implants subjected to the passivation reaction using the PEO method, it was noticed that with the increase in the process voltage, the electrode potential shifted towards a positive value. For an electrolysis voltage of 235 V, the lowest potential value of the modified surface of the magnesium implant was −1.44 V. As the process voltage increased above 235 V, the value of the potential also increased, indicating the formation of a layer without such good anti-corrosion properties on the implant surface. Corrosion current density values confirmed these results. For the Mg_03 sample, the lowest corrosion current values were obtained, which proves that the obtained sample achieved the highest electrochemical resistance to the corrosive environment. All samples were then incubated in SBF solution and tested at 37 °C for 7 days. When analyzing the values of the potentials measured for the implants after incubation in SBF, we found that the potential values for all samples had shifted towards positive potentials, which proves an additional increase in the anti-biocorrosive properties of the sample. A clear trend could be seen: with the increase in the voltage at which the implant sample was made, there was a linear decrease in the value of the electrode potential. For the Mg_03 sample, after incubation in SBF, a value of −0.75 V was obtained, representing a change of about 1.15 V in relation to the pure magnesium surface. This sample was also characterized by the lowest corrosion current density. The electrochemical analysis showed that it is most beneficial to carry out the PEO process with the proposed electrolyte composition at 235 V, as it contributed to obtaining surfaces with the best anti-biocorrosion properties.

**Figure 20:**
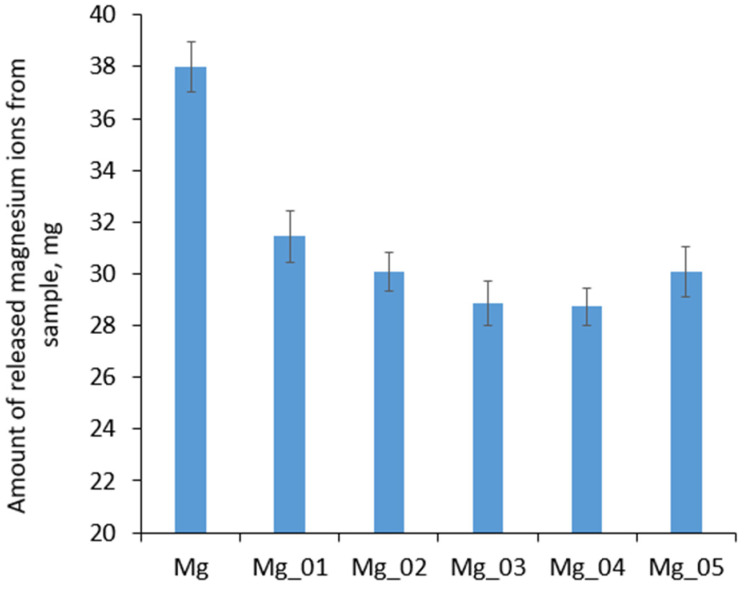
Biodegradation of the evaluated samples after 1 week.

Biodegradation is a parameter which, apart from excellent osteointegration and mechanical durability, makes magnesium a unique raw material for the preparation of metallic implants. Mg biomaterials undergo corrosion followed by sorption due to various processes carried out by host cells. However, uncontrolled or too-fast degradation may cause immunotoxin effects, inflammatory reactions, or even pain. Therefore, to prevent undesired effects from occurring prior to implantation, Mg-based medical devices must be appropriately adjusted in terms of biocorrosion resistance and reactivity with components of body fluids. Figure 20 shows the results of the biodegradation study carried out for 7 days under simulated body-like conditions (SBF, 37 °C, 5% CO_2_, high humidity). It can be observed that, after one week, initialization of biodegradation occurred, since Mg^2+^ ions began to be released into the SBF medium. By comparing the amounts of Mg^2+^ in all evaluated samples, it can be seen that, as shown in Figure 20, surface modification via PEO process resulted in a decrease in susceptibility to biocorrosion under human-like conditions, since all the prepared samples exhibited higher resistance to SBF. In the case of samples Mg_03 and Mg_04, increases of almost double the original values were noticed. Similar, yet slightly worse, results were obtained for samples Mg_01, Mg_02, and Mg_05. These results correspond to the data presented in Figure 17, Figure 18 and Figure 19, and are correlated with SEM cross-section microphotographs; these revealed the formation of MgO layers with three-layered structures of various thickness and compactness values. Our improvements to the stability of the potential implants in simulated body fluids undoubtedly proves that the appropriate adjustment of PEO process parameters enables the obtainment of biomaterials with the desired biocorrosion/biodegradation properties. This pathway of Mg-based implant modification can help to reduce the immune response of the host system after implantation and increase the success rate of tissue regeneration.

**Figure 21:**
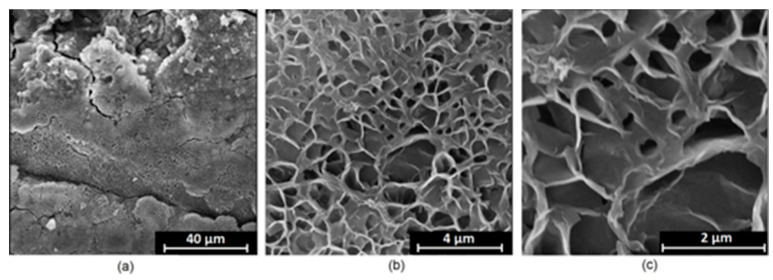
Surface appearance after incubation with SBF (sample Mg_01). Magnification: (**a**)—10,000×; (**b**)—100,000×; (**c**)—250,000×.

**Figure 22:**
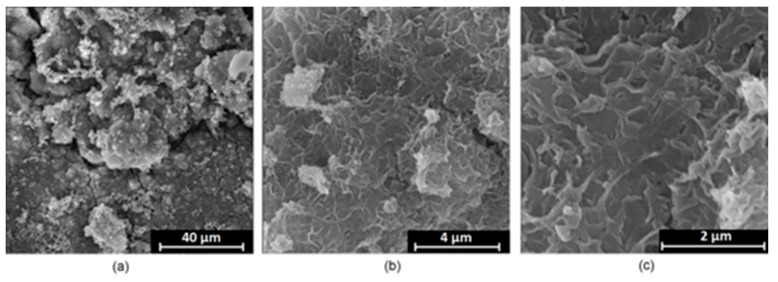
Surface appearance after incubation with SBF (sample Mg_02). Magnification: (**a**)—10,000×; (**b**)—100,000×; (**c**)—250,000×.

**Figure 23:**
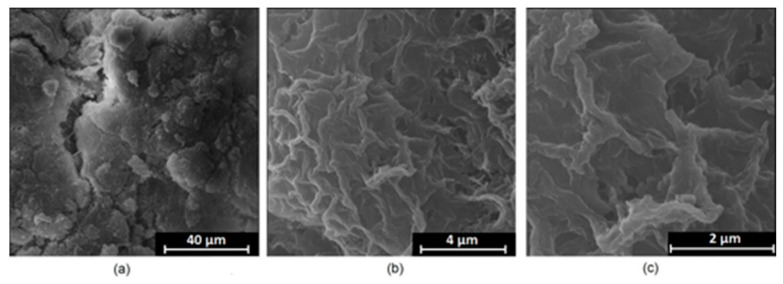
Surface appearance after incubation with SBF (sample Mg_03). Magnification: (**a**)—10,000×; (**b**)—100,000×; (**c**)—250,000×.

**Figure 24:**
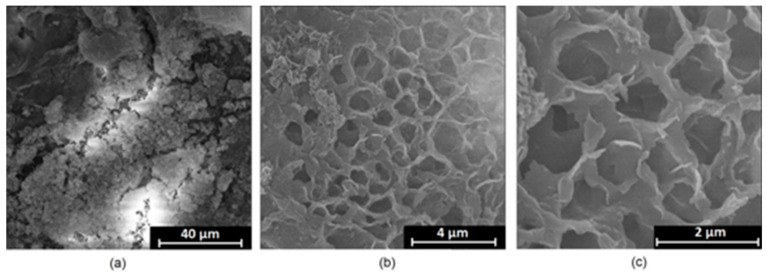
Surface appearance after incubation with SBF (sample Mg_04). Magnification: (**a**)—10,000×; (**b**)—100,000×; (**c**)—250,000×.

**Figure 25:**
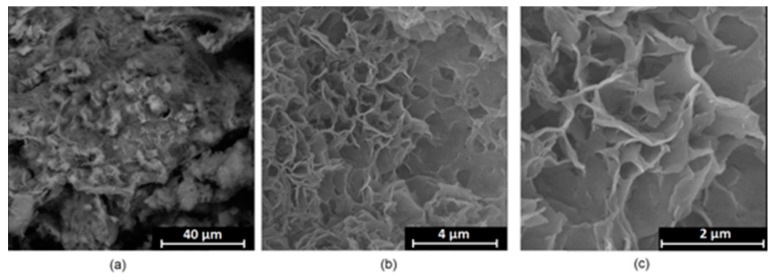
Surface appearance after incubation with SBF (sample Mg_05). Magnification: (**a**)—10,000×; (**b**)—100,000×; (**c**)—250,000×.

**Figure 26:**
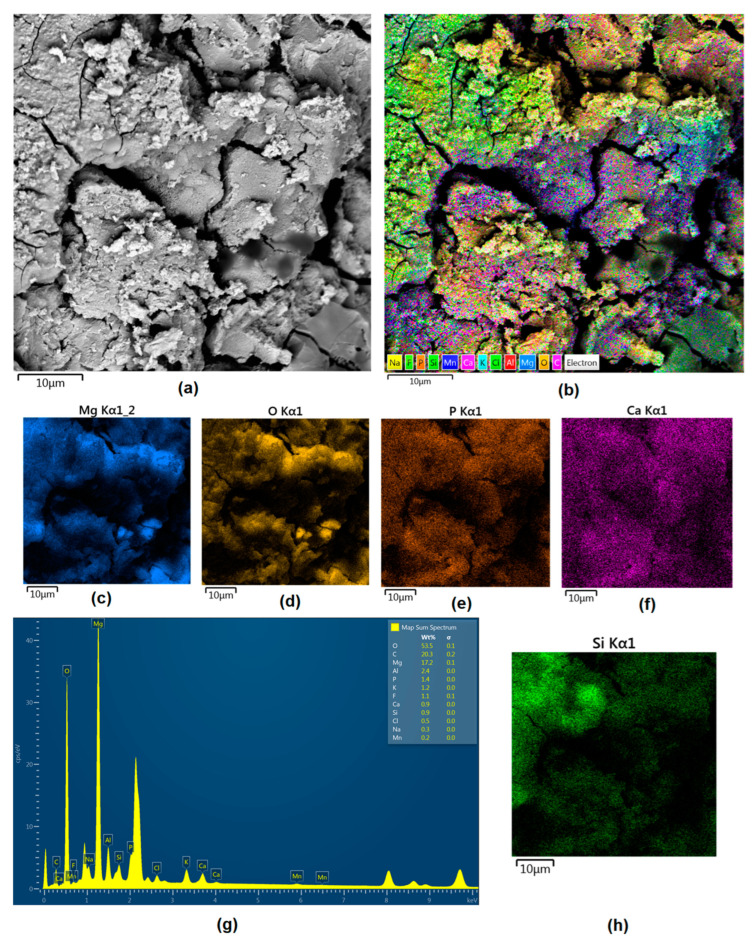
(**a**)—Surface appearance after incubation with SBF (sample Mg_01); (**b**)—surface mapping (all elements); (**c**)—magnesium; (**d**)—oxygen; (**e**)—phosphorous; (**f**)—calcium; (**g**)—element contents; (**h**)—silica.

**Figure 27:**
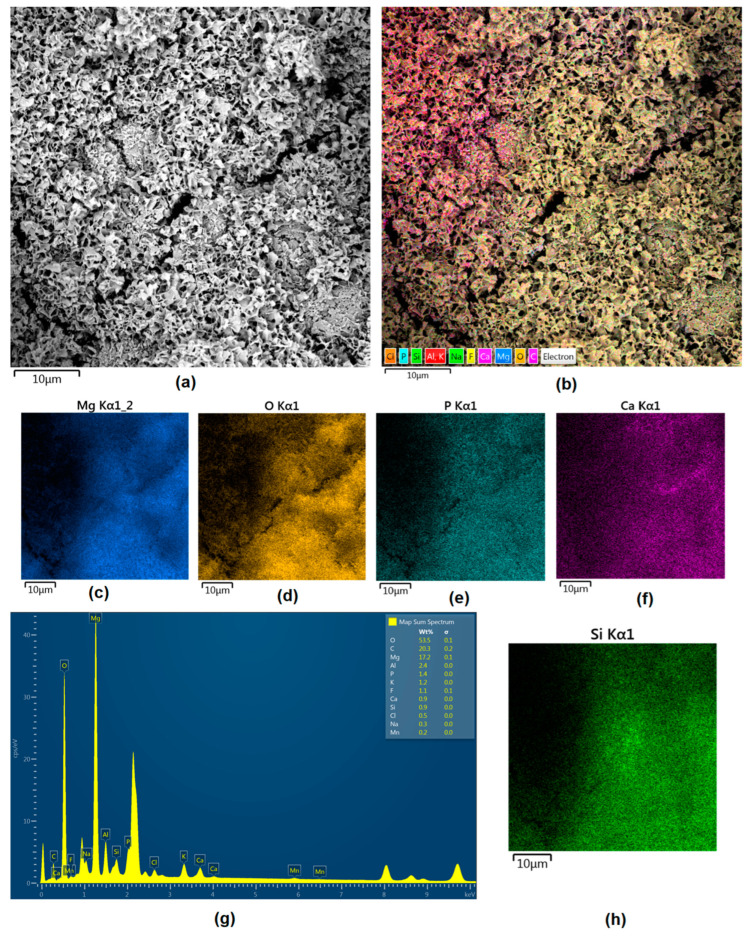
(**a**)—Surface appearance after incubation with SBF (sample Mg_02); (**b**)—surface mapping (all elements); (**c**)—magnesium; (**d**)—oxygen; (**e**)—phosphorous; (**f**)—calcium; (**g**)—element contents; (**h**)—silica.

**Figure 28:**
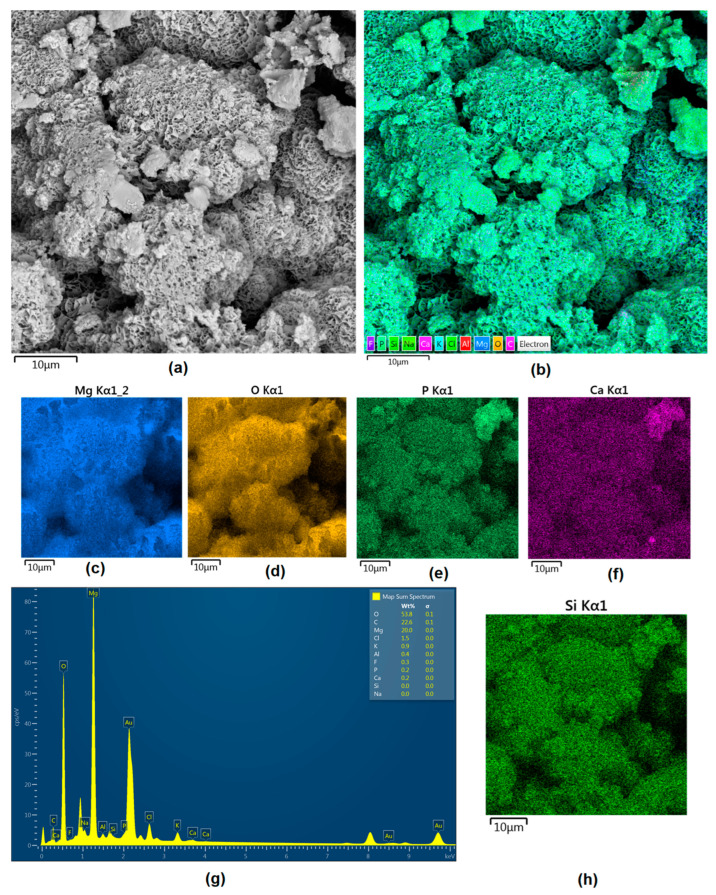
(**a**)—Surface appearance after incubation with SBF (sample Mg_03); (**b**)—surface mapping (all elements); (**c**)—magnesium; (**d**)—oxygen; (**e**)—phosphorous; (**f**)—calcium; (**g**)—element contents; (**h**)—silica.

**Figure 29:**
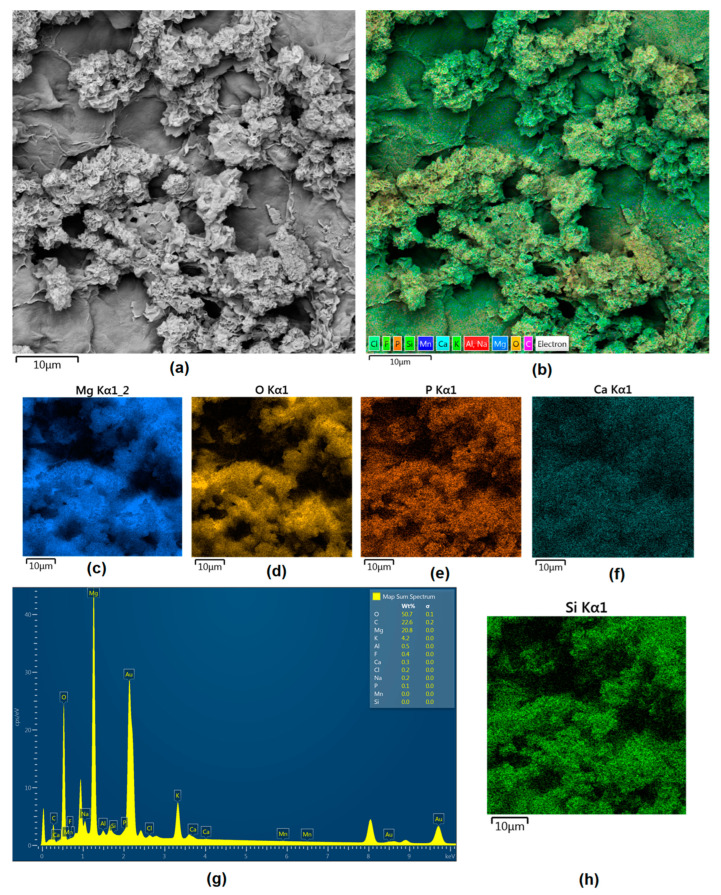
(**a**)—Surface appearance after incubation with SBF (sample Mg_4); (**b**)—surface mapping (all elements); (**c**)—magnesium; (**d**)—oxygen; (**e**)—phosphorous; (**f**)—calcium; (**g**)—element contents; (**h**)—silica.

**Figure 30:**
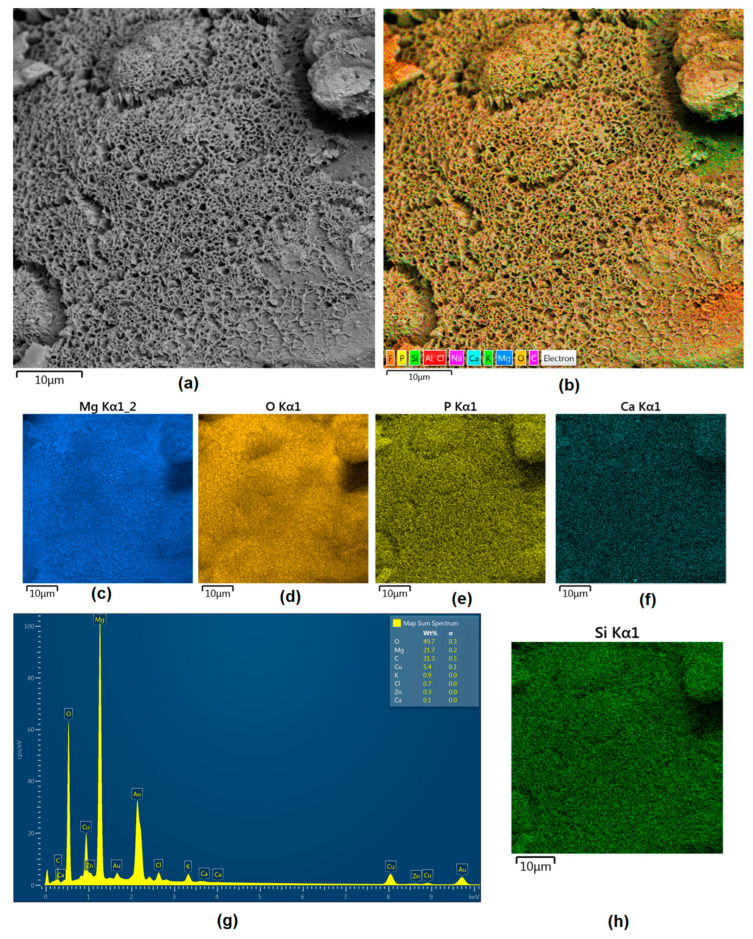
(**a**)—Surface appearance after incubation with SBF (sample Mg_01); (**b**)—surface mapping (all elements); (**c**)—magnesium; (**d**)—oxygen; (**e**) phosphorous; (**f**) calcium; (**g**) element contents; (**h**)—silica.

Mg-based biomaterials are dedicated to bone tissue regeneration applications due to the high similarity of their in mechanical properties, osteoconductive properties, biodegradability, and biocompatibility. Bone is composed of two main phases: organic (mainly collagen fibers) and inorganic (calcium phosphates and hydroxyapatite). Mineral matter is mainly responsible for tissue-supportive functions. During bone regeneration, one of the crucial processes is biomineralization. The formation of apatite structures on the biomaterial increases cellular responses and promotes bone regeneration, providing appropriate conditions for cell adhesion and proliferation as well as extracellular matrix formation. Figure 21, Figure 22, Figure 23, Figure 24 and Figure 25 present the morphologies of the samples after incubation in SBF for a 7-day period. In all cases, biomineralization occurred, which proves that the developed Mg-based implants promoted inorganic bone-like matter formation. It can be noticed that, for all samples, inorganic coatings covered the whole surface. Moreover, SEM microimages taken under magnifications of 100,000× and 250,000× revealed the highly porous nature of the samples, and that the coatings were multi-layered. Newly formed coatings exhibited mostly crystalline characteristics. By comparing the appearances of surfaces under different magnifications, it could be observed that biomineralization was still in progress [39]. In each case, pore diameters varied between 1 and 2 μm. To further investigate the effect of incubation, all samples were investigated regarding their superficial composition (Figure 26, Figure 27, Figure 28, Figure 29 and Figure 30). Consequently, the accumulated elements were revealed to be calcium and phosphorous, which uniformly covered all samples in mesh-like macrostructures. In addition, MgO and Mg_2_SiO_4_ were still visible. For the Mg_01 and Mg_02 samples, the Ca/P ratio was 0.64, which corresponded to monocalcium phosphate. The Mg_03 sample had a Ca/P 1:1 ratio, which is typical for dicalcium phosphates. The Mg_04 and Mg_05 samples were characterized by the highest ratios, suggesting that, for these implants, the formation of hydroxyapatite was observed.

**Figure 31:**
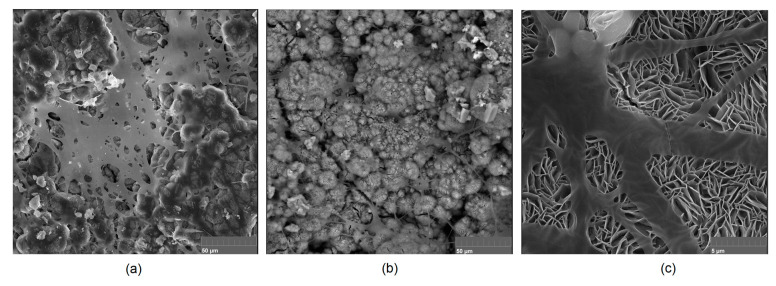
Mg_03 sample’s appearance after 7 days of cell culture: (**a**)—magnification 5000×; (**b**)—magnification 10,000×; (**c**)—magnification 20,000×.

**Figure 32:**
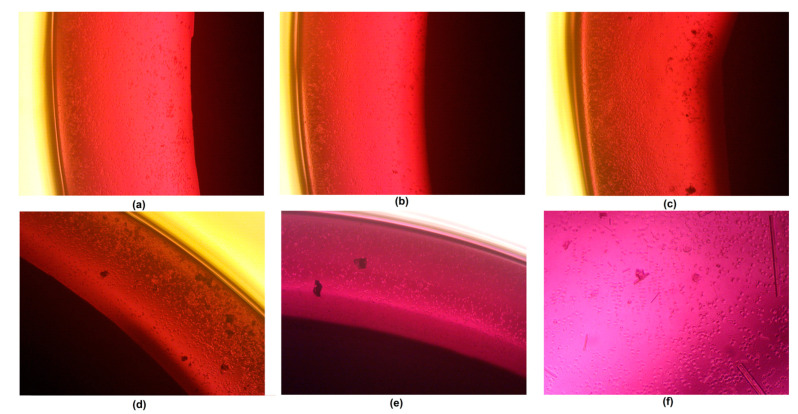
Direct contact between samples and the Mg-63 cell line: (**a**)—sample Mg_01; (**b**)—sample Mg_02; (**c**)—sample Mg_03; (**d**)—sample Mg_04; (**e**)—sample Mg_05; (**f**)—pure magnesium.

**Figure 33:**
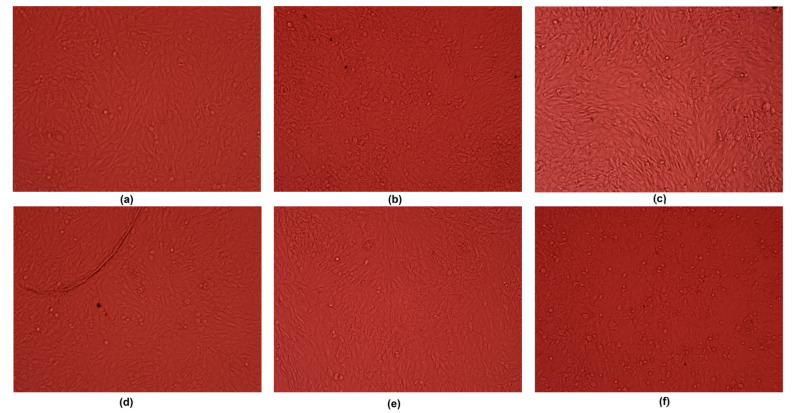
Cells’ appearances after 7 days of Mg-63 culture: (**a**)—sample Mg_01; (**b**)—sample Mg_02; (**c**)—sample Mg_03; (**d**)—sample Mg_04; (**e**) sample Mg_05; (**f**)—pure magnesium.

Biocompatibility is the most important feature of potential biomaterials; it determines it applicability in the medical/dental fields. Potential implants cannot be cytotoxic, as they need to host cells, and cannot cause allergic or inflammatory reactions. For all metallic medical devices, this parameter is strongly correlated with resistance to corrosion. It is well known that magnesium has bioactive properties and promotes bone regeneration, since it directly affects cellular behaviors and positively affects the formation of hydroxyapatite crystals [40]. Nevertheless, pure magnesium strongly reacts with SBF components, which leads to the formation of gaseous hydrogen and magnesium hydroxide, followed by rapid pH increase. Figure 31 shows the results of an MG-63 osteoblast cell culture in the presence of newly developed samples. As a control, pure magnesium was used. During the study, implants (1.9 mm in diameter) were placed in 12-hole plates (Figure 32). After first 30 min, a pH increase from 7.4 to 8.00 was observed, whereas for the pure magnesium, the pH reached almost 9. Additionally, in the case of pure Mg, hydrogen bubbles on the sample surface were formed, which confirmed the occurrence of the corrosion process. After 48 h, it could be noticed that most of the cells were attached to the surface, yet rounded, and contact inhibition of growth took place. However, for samples Mg_03 and Mg_04, osteoblasts with normal, flattened, spindle-like shapes were spotted, and these proliferated. For this reason, the experiment was repeated by placing samples in a petri dish 60 mm in diameter (Figure 33). After 7 days of the cell culture, for all evaluated implants, uniform monolayers of osteoblasts with regular morphologies and no inter-plasmatic grains connected with the extracellular matrix were obtained. It could be observed that, for pure magnesium, some osteoblasts with rounded shapes were present, indicating mediocre cytotoxic effects correlated with constant, visible H_2_ release and MgOH_2_ deposition. This led to a pH increase from 7.4 to 8.5, which was moderated by cell culture medium exchange.

**Figure 34:**
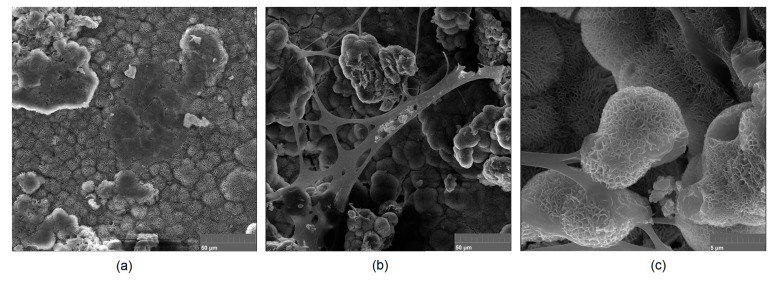
Mg_04 sample’s appearance after 7 days of cell culture: (**a**)—magnification 5000×; (**b**)—magnification 10,000×; (**c**)—magnification 20,000×.

To verify the bioactivity of the prepared samples, the implants for which the highest biocompatibility was observed were further investigated regarding their superficial appearance after an MG-63 cell culture was performed on their surfaces [41]. The results presented in Figure 31 (Mg_03) and Figure 34 (Mg_04) clearly demonstrate that the evaluated samples were not only non-cytotoxic, but also bioactive, since they promoted the extracellular formation of Mg-based surfaces, confirming the presence of hydroxyapatite crystals and calcium apatite. Moreover, newly formed collagen fibers were visible together with numerous HA crystallization points. In addition, some osteoblasts were present, but covered with organic fraction. These results reveal that Mg^2+^ ions released during the cell culture positively affected the cellular responses associated with, e.g., BMF secretion leading to bone tissue formation [42,43,44,45,46,47].

## 4. Conclusions

The aim of this study was to deeply investigate the possibility of obtaining Mg-based dental implants with functionalized surfaces using a facile, large-scale method. During the research, the PEO technique was used. For the modification, industrial equipment was applied to verify our ability to upscale the production process. The prepared products were extensively investigated regarding their crucial properties for potential application as medical devices for implantation. In this research, we confirmed that, by adjusting the process parameters, it is possible to obtain magnesium biomaterials in a one-step, facile, and rapid way. These biomaterials had properties superior to those of pure Mg and other metallic implants, and they were obtained in a manner that would enable their line production for commercial designation in the field of dentistry. The results showed that the application of the correct voltage in correlation with electrolyte composition enabled the formation of compact, thick layers that not only successfully hampered biocorrosion, which was decreased by twice as much compared to pure Mg, but also provided appropriate conditions for MG-63 cell adhesion, spread, and proliferation. Moreover, we succeeded in increasing the microhardness of the implants without decreasing other crucial parameters, such as porosity. The formation of MgO/Mg_2_SiO_4_ coatings was confirmed by FT-IR, XRD, XRF, and microscopic methods. Using SEM techniques, we extensively studied the morphological changes of pure magnesium that appeared as a consequence of PEO treatment. The cross-sections and elemental compositions of the samples have been verified. As a result of the incubation study, we confirmed the occurrence of the biomineralization process and the formation of apatite structures. Moreover, we proved that the newly developed samples did not exhibit cytotoxic effects in direct contact with osteoblast-like cells. To further explore the suitability of novel biomaterials in the field of dentistry applications, we investigated the surfaces of the implants using SEM after 7 days of MG-63 cell culture. Due to the extraordinary results of in vitro studies, our future work will focus on long-term cell–biomaterial interactions and in vivo studies of small animal models. We especially intend to investigate the impact of newly developed biomaterials on host tissues, especially immune system responses, as well as the effect on the bone defect healing process compared to traditionally applied titanium-based dental implants.

## Figures and Tables

**Table 1:** Samples description.

Sample	Description
Mg	Unmodified magnesium
Mg_01	200 V/2 A
Mg_02	225 V/2 A
Mg_03	235 V/2 A
Mg_04	250 V/2 A
Mg_05	260 V/2 A

## Data Availability

Data are available upon request.
